# Burden of Congenital and Hereditary Anomalies in Hazara Population of Khyber Pakhtunkhwa, Pakistan

**DOI:** 10.12669/pjms.38.5.5486

**Published:** 2022

**Authors:** Anisa Bibi, Syeda Farwa Naqvi, Amman Syed, Shah Zainab, Khadija Sohail, Sajid Malik

**Affiliations:** 1 Anisa Bibi, M.Phil, Human Genetics Program, Department of Zoology, Quaid-i-Azam University, Islamabad, Pakistan; 2 Syeda Farwa Naqvi, M.Phil, Human Genetics Program, Department of Zoology, Quaid-i-Azam University, Islamabad, Pakistan; 3 Amman Syed, M.Phil, Human Genetics Program, Department of Zoology, Quaid-i-Azam University, Islamabad, Pakistan; 4 Shah Zainab, M.Phil, Government Postgraduate College, Haripur, Pakistan; 5 Khadija Sohail, M.Phil, Government Postgraduate College, Haripur, Pakistan; 6 Sajid Malik, PhD, Human Genetics Program, Department of Zoology, Quaid-i-Azam University, Islamabad, Pakistan

**Keywords:** descriptive epidemiology, genetic disorders, birth defects, neurological disorders, limb anomalies

## Abstract

**Background and Objectives:**

In Pakistan, there is high incidence of congenital and hereditary anomalies (CA) which are a leading cause of infant mortality and morbidity. In order to elucidate the burden and biodemographic correlates of CA, this study was aimed to report the prevalence-pattern and phenotypic attributes of CA in the Hazara population of Khyber Pakhtunkhwa, Pakistan.

**Methods:**

In a retrospective cross-sectional study, subjects/families with CA were recruited from district hospitals and community centers. Phenotypic and descriptive data were obtained; pedigrees were analyzed and parental and biodemographic attributes were recorded.

**Results:**

A total of 1,189 independent subjects and/or families with CA were ascertained. The malformations were grouped into nine major and 95 minor categories. Neurological disorder had the highest representation (n=486; proportion=0.409; 95% CI=0.381-0.437), followed by limb defects (n=292; proportion=0.246, 95% CI=0.221-0.270), musculoskeletal defects, sensorineural/ear defects, blood disorders, eye/visual impairments, ectodermal anomalies, and congenital heart defects. In this cohort, sporadic cases were 65% and familial 35%. Parental consanguinity was significantly higher in isolated cases compared to syndromic, and in familial cases compared to sporadic. Further, speech apraxia and epilepsy were most common associations among the syndromic cases. The assessment of variables like demography, parental consanguinity, familial/sporadic nature, and pedigree structures showed conspicuous heterogeneity among the major and minor categories of CA.

**Conclusions:**

The trend of CA and high incidence of sporadic cases observed in this cohort indicate that nongenetic factors may play a significant role in their etiology which could be minimized by improving the healthcare system.

## INTRODUCTION

Congenital and hereditary anomalies (CA) are the birth abnormalities of structure, function or metabolism that occur in developmental periods.^1^ With the advancement in the control of infectious diseases, improvement of the healthcare system, hygiene and nutrition, CA have emerged as the main source of morbidity and mortality. The global prevalence of CA has been estimated to be 4%-5%.^2^,^3^ The burden of CA is very high in Pakistan due to various reasons including high rate of consanguineous unions, large sibships, low socio-economics and maternal factors. Here, an estimated 6%-9% of perinatal deaths are attributed to CA.^4^-^5^ There are a number of etiological factors underlying CA which could be summarized as genetic, maternal conditions, environmental and unknown factors.^3^,^6^-^7^

In Pakistan, the majority of the masses reside in rural areas where the healthcare infrastructure is inadequate.^8^ Hence, CA render extra burden on the low-resource healthcare system. Towards this end, a population-based study was carried out in order to elucidate the burden and prevalence-patten of CA in the young and adult Hazara population of Pakistan.

## METHODS

### Study design and sampling area:

A clinico-epidemiological study was carried out in Hazara division of Khyber-Pakhtunkhwa, Pakistan (www.pbs.gov.pk/). In a retrospective cross-sectional study design, the subjects and families with CA were recruited from District Headquarter Hospitals and special education centers during July 2018-Mar. 2021. Cases were also ascertained by visiting public places and community centers.

### Ethical consideration:

The study was approved by the Ethical Review Committee of Quaid-i-Azam University, Islamabad.(As-1070 July 8, 2015) All the data were acquired after informed consent according to Helsinki-II declaration. We used the Strengthening the Reporting of Observational Studies in Epidemiology (STROBE) statement cross-sectional reporting guidelines.^9^

### Classification of anomalies, statistical analyses:

All the index cases were physically examined and diagnosed by the resident medical officers and/or specialized doctors. Pre-diagnosed cases registered at disability and rehabilitation centers were included. Participants belonging to the remote area were brought to the nearest district hospital for clinical examination. A detailed pedigree was constructed in each case. Only the index subject in each family was included in primary data analyses.

Anomalies with traumatic or infectious nature were excluded. Index cases were categorized on the basis of gender, familial/sporadic nature, and isolated/syndromic presentations. The following order was adopted for the classification of syndromic cases: neurological disorders, musculoskeletal defects, eye/visual impairments, sensorineural/ear anomalies, and limb defects. The definition of CA was based on a standard coding system of the International Classification of Diseases and Related Health Problems (ICD-10)^6^ and the corresponding definitions were identified in OMIM (www.omim.org) and Orphanet (www.orpha.net) databases.

Categorical variables were summarized; Chi-square and Fisher-exact test statistics were applied to check the significance of distribution and *P* < 0.05 was used as the cutoff for significance. For the CA, proportions and corresponding 95% confidence intervals (CI) were calculated.

## RESULTS

### Sample characteristics:

A total of 1189 independent index cases were recruited, and the CA classified into nine major categories. Among the index cases, 678 (57%) were males (Table-I). The sporadic occurrence was more prominent compared to the familial nature (n=769 (65%) vs. n=420 (35%); respectively). Among all families, the total number of affected subjects was 2212 (1284 males, 928 females; P=0.0005).

**Table I:** Major categories of CA, familial/sporadic nature, and total number of affected family members.

Major category	Index subject			Proportion	95% CI	Familial/sporadic nature*		Total number of affecteds in all families*		
	
	Male	Female	Total			Familial	Sporadic	Males	Female	Total
Neurological disorders	276	210	486	0.409	0.381-0.437	118	368	396	301	697
Limb defects	163	129	292	0.246	0.221-0.270	102	190	336	216	552
Musculoskeletal defects	63	43	106	0.089	0.073-0.105	54	52	136	129	265
Sensorineural/ear defects	66	35	101	0.085	0.069-0.101	52	49	137	83	220
Blood disorders	48	27	75	0.063	0.049-0.077	29	46	83	34	117
Eye/visual impairments	18	21	39	0.033	0.023-0.043	23	16	57	46	103
Ectodermal anomalies	14	16	30	0.025	0.016-0.034	22	8	70	42	112
Congenital heart defects	16	10	26	0.022	0.014-0.030	6	20	24	17	41
Others	14	20	34	0.029	0.019-0.038	14	20	45	60	105
Total	678	511	1,189	1.000	-	420	769	1284	928	2212

* Chi-test statistics were statistically significant.

In the gender-wise data, sensorineural/ear defects, blood disorders and congenital heart defects were more prevalent among the male subjects (65%, 64% and 62%, respectively), while eye/visual impairments, ectodermal anomalies, and ‘Others’ category were more prevalent among the index females (54%, 53% and 59%, respectively) (Table-I). Demographic attributes of the index cases are shown in Table-II.

**Table II:** Demographic distribution of index subjects.

Variables	Male, No. (%)	Female, No. (%)	Total, No. (%)
***Age intervals (years)***			
Up-to 9	321 (47)	231 (45)	552 (47)
>9	357 (52)	280 (55)	637 (53)
Total	678 (57)	511 (43)	1189 (100)
***District***			
Haripur	307 (45)	241 (47)	548 (46)
Mansehra	191 (28)	128 (25)	319 (27)
Abbotabad	126 (19)	103 (20)	229 (19)
Kohistan	31 (5)	22 (4)	53 (5)
Batagram	23 (3)	17 (3)	40 (3)
***Mother tongue***			
Hindko	471 (69)	370 (72)	841 (71)
Pashto	83 (12)	55 (11)	138 (11)
Punjabi	56 (8)	28 (5)	84 (7)
Urdu	39 (6)	34 (7)	73 (6)
Others	31 (5)	24 (5)	55 (4)
***Caste-system***			
Awan	162 (24)	129 (25)	291 (24)
Pathan	66 (10)	60 (12)	126 (11)
Gujjar	67 (10)	43 (8)	110 (9)
Tanoli	37 (5)	28 (5)	65 (5)
Swati	30 (4)	30 (6)	60 (5)
Others	316 (47)	221 (44)	537 (45)

Chi-distribution was statistically not significant in all variables

### Classification of congenital anomalies:

The CA were resolved into nine major and at least 95 minor categories (Table-I, III). Among the major categories, neurological disorders were most frequent (n=486; 40.9%), followed by limb defects (24.6%), musculoskeletal defects (8.9%), sensorineural/ear defects (8.5%), blood disorders (6.3%), eye/visual impairments (3.3%), ectodermal anomalies (2.5%), congenital heart defects (2.2%), and Others (2.9%) (Table-I).

**Table III:** Major and minor categories of congenital/hereditary anomalies.

Major/minor categories	Frequency	Proportion	95% CI	ICD-10	OMIM
***Neurological disorders***	**486**	**0.409**	**0.381-0.437**		
Intellectual disability	176	0.148	0.128-0.168	F79	
Cerebral palsy	148	0.124	0.106-0.143	G80.0	
Epilepsy	41	0.034	0.024-0.045	G40	117100
Autism/low IQ	25	0.021	0.013-0.029	F84.0	
Down syndrome	18	0.015	0.008-0.022	Q90	190685
Hydrocephaly	14	0.012	0.006-0.018	G91.9	236600
Microcephaly	14	0.012	0.006-0.018	Q02	251200
Global developmental delay	13	0.011	0.005-0.017	Z13.42	618330
Spina bifida	11	0.009	0.004-0.015	Q05	182940
Ataxia	7	0.006	0.002-0.010	R27.0	160120
Migraine	5	0.004	0.001-0.008	G43	
Multiple sclerosis	4	0.003	0.000-0.007	G35	
Neuropathies	4	0.003	0.000-0.007	G60.9	162400
Macrocephaly	3	0.003	0.000-0.005	Q75.3	153470
Arnold Chiari malformation	1	0.001	-0.001-0.002	Q07.0	207950
Cystic encephalomalacia	1	0.001	-0.001-0.002		
Tremor	1	0.001	-0.001-0.002	R25.1	190300
***Limb defects***	**292**	**0.246**	**0.221-0.270**		
Talipes	141	0.119	0.100-0.137	Q66.0	119800
Polydactyly, postaxial	34	0.029	0.019-0.038	Q69	174200
Polydactyly, preaxial	31	0.026	0.017-0.035	Q69.1	174400
Transverse limb amputations	23	0.019	0.012-0.027	Y83.5	
Syndactyly	18	0.015	0.008-0.022	Q70	609815
Brachydactyly	10	0.008	0.003-0.014	Q68.81	113000
Clinodactyly	9	0.008	0.003-0.012	Q74.0	148520
Camptodactyly	7	0.006	0.002-0.010	Q74.0	114200
Leg length discrepancy	4	0.003	0.000-0.007	M21.7	
Constriction band syndrome	3	0.003	0.000-0.005	Q79.8	217100
Thumb hypoplasia/aplasia	3	0.003	0.000-0.005		188100
Clubbing of digits	2	0.002	-0.001-0.004	R68.3	119900
Hallux valgus	2	0.002	-0.001-0.004	M20.1	
Fibular hypoplasia	1	0.001	-0.001-0.002	Q73	
Macrodactyly	1	0.001	-0.001-0.002	Q74.2	155500
Radial hemimelia	1	0.001	-0.001-0.002	Q73.8	
Symphalangism	1	0.001	-0.001-0.002	Q70.9	185800
Trigger thumb	1	0.001	-0.001-0.002	M65.319	190410
***Musculoskeletal defects***	**106**	**0.089**	**0.073-0.105**		
Muscular dystrophy	23	0.019	0.012-0.027	G71.0	310200
Hypotonia (limbs)/myopathies	23	0.019	0.012-0.027	P94.2	300868
Dwarfisms	20	0.017	0.010-0.024	E34.3	100800
Congenital hip dysplasia	11	0.009	0.004-0.015	Q65.8	142700
Scoliosis	6	0.005	0.001-0.009	M41	181800
Kyphoscoliosis	4	0.003	0.000-0.007	M40	610170
Osteogenesis imperfecta	4	0.003	0.000-0.007	Q78.0	166200
Arthrogryposis	2	0.002	-0.001-0.004	Q74.3	108120
Carpal fusion	2	0.002	-0.001-0.004		
Exostosis	2	0.002	-0.001-0.004	Q78.6	133700
Klippel-Feil syndrome	2	0.002	-0.001-0.004	Q76.1	118100
Pectus carinatum	2	0.002	-0.001-0.004	Q67.7	
DuPan syndrome	1	0.001	-0.001-0.002		228900
Genu valgum	1	0.001	-0.001-0.002	M21.06	137370
Muscular torticollis	1	0.001	-0.001-0.002	M43.6	189600
Rheumatoid arthritis	1	0.001	-0.001-0.002	M06	180300
Rickets, vitamin-D resistant	1	0.001	-0.001-0.002	E83.3	277440
***Sensorineural/ear defects***	**101**	**0.085**	**0.069-0.101**		
Deaf and mute	88	0.074	0.059-0.089	H91.3	304500
Microtia/deformed pinna	8	0.007	0.002-0.011	Q17.2	600674
Speech apraxia	3	0.003	0.000-0.005	R47.9	602081
Deaf only	1	0.001	-0.001-0.002		
Mute only	1	0.001	-0.001-0.002		
***Blood disorders***	**75**	**0.063**	**0.049-0.077**		
Thalassemia	59	0.050	0.037-0.062	D56	613985
Hemophilia	15	0.013	0.006-0.019	D66	306700
Fanconi anemia	1	0.001	-0.001-0.002	D61.09	227650
***Eye/visual impairments***	**39**	**0.033**	**0.023-0.043**		
Blindness	20	0.017	0.010-0.024	H54	216900
Squint/strabismus	9	0.008	0.003-0.012	H50.9	185100
Colour blindness	3	0.003	0.000-0.005	H53.5	303800
High myopia	3	0.003	0.000-0.005	H52.10	
Night blindness	3	0.003	0.000-0.005	H53.60	310500
Anophthalmia	1	0.001	-0.001-0.002	Q11.2	251600
***Ectodermal anomalies***	**30**	**0.025**	**0.016-0.034**		
Atopic dermatitis/eczema	8	0.007	0.002-0.011	L20	603165
Albinism, oculocutaneous	5	0.004	0.001-0.008	E70.3	203100
Alopecia totalis	4	0.003	0.000-0.007	L63.0	203655
Psoriasis	3	0.003	0.000-0.005	L40	177900
Ectodermal dysplasia	2	0.002	-0.001-0.004	Q82.4	305100
Hypotrichosis	2	0.002	-0.001-0.004	Q84.0	605389
Ichthyosis	2	0.002	-0.001-0.004	L85.0	242300
Alopecia areata	1	0.001	-0.001-0.002	L63	104000
Neurofibromatosis	1	0.001	-0.001-0.002	Q85.0	162200
Onychodystrophy	1	0.001	-0.001-0.002	L60.3	161050
Palmoplantar keratoderma	1	0.001	-0.001-0.002	L40.3	144200
***Congenital heart defects***	**26**	**0.022**	**0.014-0.030**		
Ventricular septal defect	12	0.010	0.004-0.016	Q21.0	614429
Arterial septal defect	6	0.005	0.001-0.009	Q21.1	108800
Coronary artery disease	5	0.004	0.001-0.008	I125.10	608901
Atrioventricular canal defect	2	0.002	-0.001-0.004	Q21.2	606215
Bradycardia	1	0.001	-0.001-0.002	R00.1	
***Others***	**34**	**0.029**	**0.019-0.038**		
Cleft lip/cleft pallet	8	0.007	0.002-0.011	Q37	119530
Bardet-Biedl syndrome	5	0.004	0.001-0.008	Q87.89	209900
Anomalies of kidney/urinary tract	5	0.003	0.000-0.005	Q64.9	
Neonatal diabetes mellitus	4	0.003	0.000-0.007	P70.2	222100
Celiac disease	2	0.002	-0.001-0.004	K90.0	212750
Congenital hypothyroidism	2	0.002	-0.001-0.004	E03.1	275200
Lymphedema	2	0.002	-0.001-0.004	I89.0	
Anorectal malformations	1	0.001	-0.001-0.002		107100
Congenital immunodeficiency	1	0.001	-0.001-0.002	D89.9	
Glucose 6-P-dehydrogenase deficiency	1	0.001	-0.001-0.002	D55.0	305900
Hirschsprung disease	1	0.001	-0.001-0.002	Q43.1	142623
Neonatal adiposity	1	0.001	-0.001-0.002	E66.9	
Orofacial anomaly	1	0.001	-0.001-0.002	G24.4	

The neurological disorders were further grouped into 17 subcategories (Table-III). Among these, the most prevalent were intellectual disability (ID; n=176), cerebral palsy (148), epilepsy (n=41), autism/low IQ (n=25), Down syndrome (n=18), hydrocephaly (n=14), and microcephaly (n=14). Limb defects were resolved into 18 separate entities (detailed distribution given in Table-III).

### Familial vs sporadic presentations and consanguinity:

Analyses of pedigree structures revealed that there were 420 familial cases (35%) while remaining 769 (65%) had sporadic presentations (P<0.0001) (Table-I). The highest representation of familial cases was in ectodermal anomalies (77%), followed by eye/visual impairments (59%), while the lowest ratio was witnessed in neurological disorders (24%) and limb defects (35%).

The parental consanguinity in this cohort was estimated to be 66%; it ranged from 60% in limb defects to 81% in congenital heart defects (P=0.07). Consanguinity was significantly higher in the familial cases compared to sporadic (72% vs. 63%, respectively; P=0.004).

## DISCUSSION

This is the first study reporting detailed clinical and descriptive epidemiological aspects of CA in the Hazara population of Pakistan. The prevalence-pattern of CA is useful implications in guiding resource allocation, management plans and therapeutic interventions.

CA related to the central nervous system (CNS) have been shown to be the most common types in many studies carried out internationally and locally.^2^,^4^,^7^ In our cohort, neurological disorders were observed to be the most prevalent (41%), followed by limb defects (25%) and musculoskeletal defects (9%). This pattern is also concordant with previous studies conducted in Pakistani populations of Lahore, Peshawar and Kurram Tribal Agency.^4^,^10^,^11^ In addition to a number of maternal, environmental and non-genetic factors, a likely reason for the high incidence of neurological disorders is that CNS requires an extended period of development and morphogenesis during embryonic development. Among the neurological disorders, intellectual disabilities (ID) were most conspicuous in this cohort. Pakistan has been identified as one of the developing countries with the highest percentage of children with ID.^12^ Certain non-genetic factors like advanced maternal age at birth, minimal maternal education, low socioeconomic status, rural origin, less availability of healthcare system, poor antenatal care, maternal malnutrition and infections contribute to the increased rate of ID in developing countries including Pakistan.^1^,^6^

Limb defects were the second largest group of CA in the present cohort (25%). An epidemiological study on CA carried out in Sialkot, Pakistan, reported that limb defects were the most prevalent group (47%).^5^ In another study conducted in Kurram Agency of Northwest Pakistan, Zahra et al. reported that limb defects were the third most common types (21%), after neurological disorders (34%) and musculoskeletal defects (23%).^11^ Many limb defects are the source of disability, i.e., talipes, transverse limb amputations, leg length discrepancy, constriction band syndrome, thumb hypoplasia/aplasia, fibular hypoplasia, radial hemimelia.

There was a high incidence of sporadic cases compared to the familial (65% vs. 35%). This observation is concordant with a recent epidemiological study carried out in Sialkot, Pakistan.^5^ In that study, the authors argued that a high preponderance of sporadic presentations among the limb and neurological disorders and a relatively reduced level of parental consanguinity may suggest a significant involvement of environmental factors in the etiology of these anomalies. Studies have shown that specific nongenetic factors may be involved in the etiology of certain types of CA. Brender and Weyer showed that there was a high risk of limb anomalies among the mothers who were exposed to agricultural compounds in water.^13^

The consanguinity rate was calculated to be 66% in our cohort and the highest rate of consanguinity evident in congenital heart defects (81%) and sensorineural/ear defects (77%). These observations are concordant with a study conducted by Zahra et al. who showed that the highest inbred unions were observed in children with congenital heart defects and deaf/mute cases.^11^ Furthermore, the familial cases had a significantly higher likelihood of parental consanguinity compared to the sporadic cases (P=0.004), which may suggest the key role of recessive genetic factors. In order to understand the more rational role of consanguinity in various CA types, it would be worthwhile to estimate the background consanguinity in the population (see for instance Rittler et al.).^14^

### Limitation of the Study:

The current study has also several limitations. For instance, this study does not report the true prevalence or incidence rate of CA, and molecular diagnosis through mutation analyses or chromosomal investigations. Further, various physiological and metabolic disorders may remain unreported.

## CONCLUSION

This study presents a comprehensive clinical and descriptive account of CA in the Hazara population of Pakistan. Neurological disorder, limb defects and musculoskeletal defects render the highest burden and comprised 74% of the sample. The pattern of anomalies and the high incidence of sporadic cases may be indicative of nongenetic etiological factors. The burden of these anomalies can be minimized by improving health education, provision of antenatal and perinatal care, premarital counselling, genetic screening and molecular diagnosis of CA, and in general strengthening the healthcare system.
